# Effectiveness of feeding different biochars on growth, digestibility, body composition, hematology and mineral status of the Nile tilapia, *Oreochromis niloticus*

**DOI:** 10.1038/s41598-024-63463-4

**Published:** 2024-06-12

**Authors:** Muhammad Amjad, Syed Makhdoom Hussain, Shafaqat Ali, Muhammad Rizwan, Khalid A. Al-Ghanim, Jean Wan Hong Yong

**Affiliations:** 1 Fish Nutrition Lab, Department of Zoology, Government College University Faisalabad, Faisalabad, 38000 Punjab Pakistan; 2 Department of Environmental Sciences, Government College University Faisalabad, Faisalabad, 38000 Punjab Pakistan; 3 Department of Biological Sciences and Technology, China Medical University, Taichung, 40402 Taiwan; 4 Department of Zoology, College of Science, King Saud University, 11451 Riyadh, Saudi Arabia; 5 Department of Biosystems and Technology, Swedish University of Agricultural Sciences, 23456 Alnarp, Sweden

**Keywords:** Biochar, Growth, Nutrient digestibility, Hematology, Mineral status, Physiology, Zoology, Environmental sciences, Health care

## Abstract

*Oreochromis niloticus* fingerlings (5.15 ± 0.02 g; n = 315) were fed with different types of biochar (BC)-supplemented sunflower meal-based (SFM) diet to investigate the effects of various BC inclusions on their nutritional digestibility, body composition, hematology and mineral status for 60 days. Seven different diets were formulated based on the SFM based diet: one was a control (TD-I, CON) and the other six diets were supplemented with 2% BC derived from different sources. These BCs were derived from the following: cotton stick (CSBC, TD-II), wheat straw (WSBC, TD-III), corn cob (CCBC, TD-IV), house waste (HWBC, TD-V), grass waste (GWBC, TD-VI), and green waste (GwBC, TD-VII) biochar. There were three replicates for each test diet. Each tank had fifteen tilapia fingerlings, and they were fed with 5% of their live wet weight and twice daily. The outcomes showed that the supplementation of CCBC significantly elevated the growth, nutrient absorption, and body composition of the *O. niloticus* fingerlings (*p* < 0.05); with concomitant lowering of the quantity of nutrients released into the water bodies whereas HWBC gave negative impacts. The maximal mineral absorption efficiency (Ca, Na, K, Cu, Fe, P, and Zn) was achieved by the supplementation of 2% CCBC. All hematological parameters showed positive improvements (*p* < 0.05) with CCBC. Interestingly, CCBC significantly improved the growth, digestibility, body composition, hematology, and mineral status of *O. niloticus*.

## Introduction

Fishes, being at the tertiary level of the food chain, provide humans with a crucial and abundant source of nutrition. The proteins, minerals, unsaturated fats, and vitamins present in fish make it an excellent dietary choice^1^.The American Heart Association stated that eating fish twice a week is enough to fulfill one’s daily needs for omega-3 fatty acids^2^.

The most significant input and a limiting component in aquaculture is the availability of feed. Commercial aqua-feeds are usually a mixture of several feed ingredients that provide the cultivated aquatic organisms with the essential nutrients that they need. Owing to their advantageous qualities, fishmeal (FM) and fish oil (FO) are regarded as the most important feed components^3^. High-quality animal protein is primarily supplied by aquaculture, which also makes a crucial role in global food safety. Owing to the increase in population, it is expected to grow further in response to rising demand^4^. More than 70% of the world's aquaculture food production comes from freshwater aquaculture^5^. In most countries, it has become an important industry due to high demand of fish and shellfish across the globe^6^. These nutritional benefits of FM make it an essential part of fish feed including polypeptide chain balance, vitamin content, digestibility, growth-promoting attributes, and attractive smell^7^. However, the prices of FM have risen over the past decade due to its increasing demand^8^. Some have voiced economic, social, and environmental issues over the use of FM as animal feed^9^. Therefore, finding an alternative, less expensive plant-based diet without affecting fish performance can be crucial to the sustainability of fish farming in the future. Sunflower meal (SFM) is now employed as a viable protein source in aquaculture diets attributed to its elevated protein content, enhanced palatability, superior digestibility and low antinutritional factors. The protein concentration ranges from 36 to 40%, with higher levels of methionine and tryptophan than other plant protein sources^10,11^. It is found that SFM may replace up to 75% of the diet of common carp without negatively impacting growth, carcass, or blood profile ^12^. Furthermore, SFM can be utilized as an alternative because it is widely available and inexpensive^13^.

The last 20 years have seen a tremendous increase in population, which has led to rapid industrialization and urbanization. Due to these factors, environmental pollution and the scarcity of  clean water has increased^14^. Toxic industrial chemicals, excessive fertilizers, heavy metals, food additives, insecticides, personal care items, and veterinary supplies (hormones), are few examples of the wide spectrum environmental pollution^15–19^. The organic wastes emit carbon dioxide, one of the greenhouse gas, into the atmosphere when they break down naturally^20^. BC is a form of carbon, formed by the pyrolysis of biomass. Thus, converting waste into biochar (BC) can sequester the carbon and reduce this greenhouse gas from environment^21,22^. Scientists are producing BC, nano-biochar, and other value-added byproducts from various wastes as feedstock^23,24^.

BC is a carbon-based substance produced by heating organic matter in an absence or presence of a low amount of oxygen^22,25–27^. It is light weight, dark in color and with a higher amount of carbon^28^. Its valuable agricultural features include improving soil structure, decreasing bulk density, boosting porosity, water retention and improving plant growth^29–32^. Moreover, its primary application is to regulate or reduce ammonia levels in animal production systems.

*Oreochromis niloticus*, commonly known as Nile tilapia, is the world's most popular fish species. Recently, there was a significant rise in the production of *Nile tilapia*. In developing countries, it is considered as a valuable species^33^. In 2020, it played an important role contributing about 5% to global aquaculture production^34^. Because of its adaptability, high meat quality, fast growth rate, and widespread farming, it is one of the most commonly farmed species in the world^35^. However, high feed costs, notably soybean meal, limit tilapia farming and especially economic viability^36^. The effects of BC on growth, body composition, digestibility, hematology and mineralization of *O. niloticus* has yet to be studied. The current investigation was carried out on *O. niloticus* to assess the efficacy of adding 2% level of the various BC to fish growth, nutrient absorption, carcass outcomes, hematology and mineral status*.*

## Materials and methods

This study was carried out in the Fish Nutrition Laboratory of the Department of Zoology at GC University in Faisalabad, Pakistan.

### Ethical statement

All research protocols were approved by the ethical guidelines of Animal Welfare and Ethics, provided by Government College University Faisalabad. The study was conducted in compliance with the ARRIVE guidelines and all methods were performed in accordance with the relevant guidelines and regulations. The authors confirm that the study had been conducted in an ethical and responsible manner.

### Experimental trial

We purchased fingerlings of *O. niloticus* from Fish Seed Hatchery, Faisalabad, Pakistan. The average size of the total 315 fishes was 5.15 ± 0.02 g. The fingerlings were brought to the lab in polythene bags with adequate aeration. After being carefully transported to circular cement containers with a capacity of 400 L, they were left undisturbed for the whole night. For 10 days at 28 °C, the fingerlings were acclimatized in an aerated environment^37^. In order to avoid detrimental inflammation or ecto-parasites, the fingerlings were immersed in a NaCl solution^38^. Commercial fish feed was given to these fingerlings twice a day.

### Production of biochar

Following crushing and drying, a variety of biomass materials were collected for the purpose of producing BC. These materials included cotton sticks, wheat straw, corncob, House waster, grass waste, and green waste. Separate batches of BC were pyrolyzed in a top-lit draft gasifier. After that, they were cooled and then ground into extremely fine particles by passing them through a 2 mm sieve^39^. Before being utilized to make the experimental diets, the powdered BC was stored in an airtight container.

### Feed Ingredients and processing

One control diet and six experimental treatments were formulated. The test diets were supplemented with a 2% BC supplement from different sources (TD-II, corn stick BC, CSBC); (TD-III, wheat straw BC, WSBC); (TD-IV, corn cob BC, CCBC); (TD-V, house waste BC, HWBC); (TD-VI, grass waste BC, GWBC); and (TD-VII, green waste BC, GwBC), whereas the control group (TD-I, CON) did not get any BC. Once all the materials had been crushed through a 0.5 mm sieve, they were well mixed for five minutes and fish oil was gradually added. Afterwards, the components were combined with a precise amount of water (10–15% w/w) to make a homogeneous dough^40^. The dough was finally run through a pelleting machine to turn into pellets. Pellets were stored at − 20 °C until they were utilized, following an oven-dried process. The Tables 1 and 2 displays the composition and proximate of feed components and experimental diets.

**Table 1:** The composition of the test diets (%).

Ingredients	CON (TD-I)	CSBC (TD-II)	WSBC (TD-III)	CCBC (TD-IV)	HWBC (TD-V)	GWBC (TD-VI)	GwBC (TD-VII)
BC (g/kg)	0	20	20	20	20	20	20
Sunflower meal	520	520	520	520	520	520	520
Fish meal	160	160	160	160	160	160	160
Wheat flour	120*	100	100	100	100	100	100
Rice Polish	90	90	90	90	90	90	90
Fish oil	70	70	70	70	70	70	70
Chromic oxide	10	10	10	10	10	10	10
Vitamin Premix*	10	10	10	10	10	10	10
Mineral premix**	10	10	10	10	10	10	10
Ascorbic acid	10	10	10	10	10	10	10

*Vitamin (Vit.) premix kg^−1^: Vit. C: 15,000 mg, Vit. B_2_: 7000 mg, Vit. A: 15,000,000 IU, Vit. B_6_: 4000 mg, Vit. D3: 3,000,000 IU, Vit. E:30,000 IU, Vit. B_12_: 40 mg, Vit. K_3_: 8000 mg, Folic acid: 1500 mg, Nicotinic acid: 60,000 mg, Ca pantothenate: 12,000 mg.

**Mineral premix kg^−1^: P: 135 g, Ca: 155 g, Mg: 55 g, Na: 45 g, Cu: 600 mg, Mn: 2000 mg, Co: 40 mg, Zn: 3000 mg, Se: 3 m, Fe: 1000 mg, I: 40 mg.

Biochar (BC), Control (CON, TD-I), cotton stick BC (CSBC, TD-II), wheat straw (WSBC, TD-III), corn cob (CCBC, TD-IV), house waste (HWBC, TD-V), grass waste (GWBC, TD-VI), and green waste (GwBC, TD-VII).

**Table 2:** The chemical makeup (%) of the various feed components and experimental diets.

Feed components	Fish meal	Rice polish	Wheat flour	Sunflower
Dry matter (%)	93.26	95.07	91.4	94.73
Crude fat (%)	6.95	11.78	2.54	3.23
Crude protein (%)	50.05	12.76	08.71	41.61
Crude fiber (%)	1.53	12.90	2.52	1.54
Ash (%)	22.17	12.21	1.67	08.74
Carbohydrates	18.67	51.25	81.23	42.3
Gross energy (GE) (kcal/g)	2.55	3.21	3.00	2.41

BC	Nutrient composition of experimental diets			
	Crude fat (%)	Crude protein (%)	Gross energy (kcal/g)	
CON	8.22 ± 0.01	30.63 ± 0.02	3.53 ± 0.02	
CSBC	8.23 ± 0.02	30.55 ± 0.04	3.53 ± 0.01	
WSBC	8.24 ± 0.01	30.67 ± 0.03	3.54 ± 0.02	
CCBC	8.24 ± 0.02	30.53 ± 0.03	3.52 ± 0.01	
HWBC	8.25 ± 0.02	30.53 ± 0.02	3.52 ± 0.02	
GWBC	8.24 ± 0.03	30.66 ± 0.02	3.53 ± 0.01	
GwBC	8.24 ± 0.03	30.65 ± 0.02	3.53 ± 0.01	

Biochar (BC), Control (CON, TD- I), cotton stick BC (CSBC, TD-II), wheat straw (WSBC, TD-III), corn cob (CCBC, TD-IV), house waste (HWBC, TD-V), grass waste (GWBC, TD-VI), and green waste (GwBC, TD-VII).

### Growth study

The *O. niloticus* fingerlings from each tank were collectively weighted at the beginning and upon completion of the growth trial. The below-mentioned standard formulae^41^ were used to compute the feed conversion ratio (FCR), weight gain percentage (WG%), and specific growth rate (SGR).

$$\text{WG }(\text{g})=(\text{Final weight}-\text{Initial weight})$$

$$\text{FCR}=\text{Total dry feed intake }(\text{g})/\text{Wet weight gain}$$

$$\text{WG\%}=(\text{Final weight}-\text{Initial weight})\times 100/\text{Initial weight}$$

$$\text{SGR}=(\text{In }(\text{Final weight})-\text{In }(\text{Initial weight})\times 100/\text{No}.\text{ of days}$$

### Chemical analysis of feed, feces, and muscle

A mortar and pestle were used to homogenize samples (1 g), taken from each tank, of the test diet and feces. The materials were evaluated by standard procedures^42,43^. For 12 h, the samples were dried in an oven at 105 °C to determine its moisture content. The petroleum ether extraction technique was utilized to extract crude fat (CF) utilizing a Soxtec HT2 1045 system. The micro Kjeldahl apparatus was utilized to estimate crude protein (CP; N*6.25), and an electric furnace lit to 650 °C for 12 h was employed to identify ash. The gross energy (GE) was measured using the oxygen bomb calorimeter. In order to generate calibrated standards for mineral estimation commercial procedures were used^44^.

### Nutrient digestibility

One-gram samples of dried feed, body and feces were pulverized and homogenized using a mortar and pestle for the determination of nutritional digestibility. The Standard technique by Brown ^45^ was used to estimate the apparent nutritional digestibility of each meal.

$$\text{Digestibility (\%)} =\text{[100}-({100}\times \frac{ \, \text{Percent marker in diet}\times \text{Percent nutrient in feces}}{ \, \text{Percent marker in feces} \times \text{Percent nutrient in diet} })]$$

### Hematological study

Following the 60-day feeding trial, three fish from each tank were dipped into 150 mg^−1^ tricane methane sulfonate for anesthesia^46^. After that, blood sample was drawn from the caudal vein by using heparinized syringe. The. A micro-hematocrit and a capillary tube were utilized to calculate the hematocrit. Platelets (PLTs), white blood cells (WBC) and red blood cells (RBCs), were carried out using an approved Neuber counting chamber^47^. Utilizing the technique established the hemoglobin level was determined^48^. The mean hemoglobin concentration (MCHC), mean corpuscular hemoglobin (MCH) and mean corpuscular volume (MCV) were determined using the formulae^49^.

### Statistical analysis

The growth parameters, blood profile, whole body composition, nutrient digestibility and mineral status data were evaluated using a one-way ANOVA^50^. The difference between means was examined using Tukey’s Honest Significant Difference Test; a p-value of less than 0.05 was considered significant^51^. Data analysis was performed using the CoStat Computer Package (version 6.303, USA).

## Results

### Growth parameters

**Figure 1:**
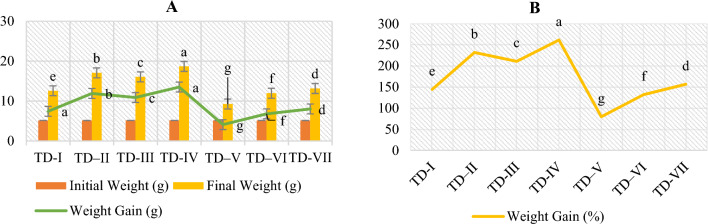
Growth parameters of *O. niloticus* fed with 2% BC supplementation (**A**) initial weight (g), final weight (g) and weight gain (WG; g), (**B**) WG (%).

Figure 1 showed the growth parameters of *O. niloticus* fed diets supplemented with 2% of various types of BC. The size of fingerlings was observed to be comparable in the current investigation. When compared to other BCs and the control diet, fish that were given a CCBC SFM-based diet (test diet IV) exhibited a highest increase in weight gain. Test diet V, which consisted of HWBC SFM-based diet, resulted in the lowest growth rate of the fingerlings compared to the other diets, indicating detrimental effect on fingerlings growth. Even the HWBC-based diet surpassed the control diet in terms of growth.

**Figure 2:**
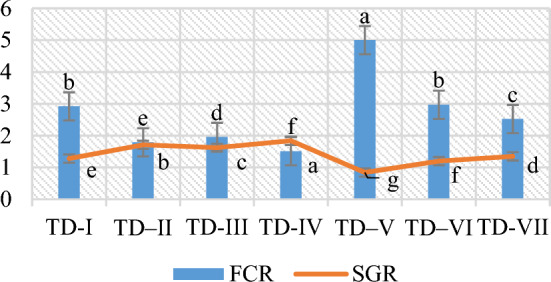
The FCR and SGR of *O. niloticus* fed with 2% BC supplementation.

The greatest weight gains (13.52 g) and weight gain percentage (261.53%) were recorded in *O. niloticus* fingerlings that were fed CCBC supplemented SFM-based diet. The supplementation of HWBC- SFM based diet resulted in detrimental effect on weight gain. The results indicated an increase in growth with the exception of HWBC compared to all other diets, including the control diet. The CCBC-based diet exhibited the optimal values for FCR and SGR (Fig. 2), with FCR of 1.51 and SGR of 1.84, respectively. The HWBC-based diet, however, had a negative effect (FCR: 2.75 SGR: 0.84).

### Nutrient digestibility

**Figure 3:**
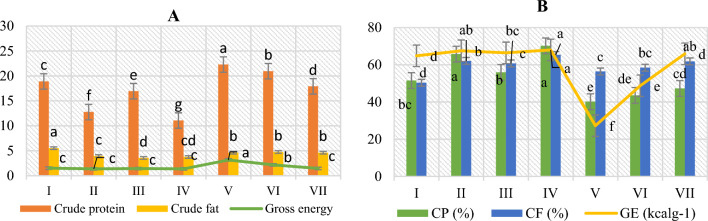
(**A**) The nutrient digestibility of feces and (**B**) Apparent digestibility coefficient of *O. niloticus* fed with 2% BC supplementation.

According to the current investigation, a CCBC-based diet resulted in the highest significant values of nutrient digestibility (Fig. 3) in feces (crude protein: 11.07%, crude fat: 3.76%, and gross energy: 1.38%) where as HWBC-based diet (crude protein: 22.28%, crude fat: 4.62%, gross energy: 3.16%) was the only test diet that demonstrated negative results in terms of nutrient digestibility of feces. These values showed that maximal amount of nutrients has been released in the feces. The highest results of ADC% (crude protein: 70.2%, gross energy: 68.02% and crude fat: 65.34%) of *O. niloticus* were observed on a CCBC-based diet. The lowest results of ADC% were seen in the HWBC-based diet (crude protein: 40.15%, gross energy: 27.27% and crude fat: 56.34%).

### Body composition

**Figure 4:**
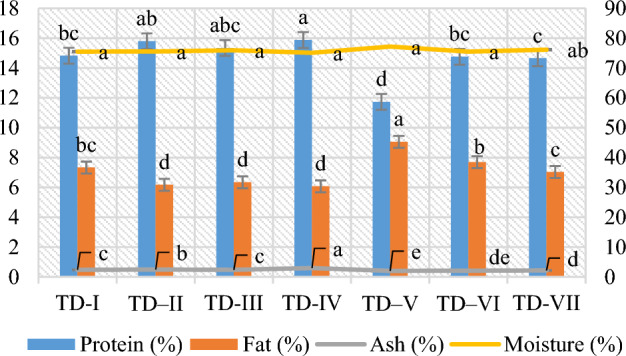
The body composition (protein%, fat%, ash% and moisture%) of *O. niloticus* fed with 2% BC supplementation.

Figure 4 showed the carcass composition of *O. niloticus* fingerlings fed diets supplemented with 2% of the various BC types. The impact of different types of BCs on carcass outcomes (protein, fat, ash, and moisture) were significant (*p* < 0.05). The proportion of fat (6.07%) and moisture (75.10%) were lower, while the values of protein (15.87%) and ash (2.96%) were significantly greater when fed with (TD-IV) CCBC-based diet. The highest fat level (9.55%) and moisture content (77.17%), and lowest protein (11.73%), and ash (2.04%) contents were found in (TD-V) containing HWBC.

### Hematology

The hematological results are displayed in Table 3 and demonstrated a significant (*p* < 0.05) improvement in Hb, PLT, RBCs, WBCs, and all other hematological indices. Notable outcomes were obtained with a 2% CCBC supplement. The results indicate that *O. niloticus* fed on a diet based on SFM and supplemented with 2% CCBC (TD-IV) had the greatest levels of PCV% (26.74), WBCs (7.56 × 10^−6^ mm^−3^), RBCs (3.42 × 10^−6^ mm^−3^), MCHC% (30.86), MCH (25.03), and MCV (85.19). The samples that included 2% HWBC (TD-V) exhibited the lowest values of Hb (4.70 g/100 ml), WBCs (5.48 × 10^−6^ mm^−3^), PCV% (15.37), MCHC% (18.45), MCH (16.34), and MCV (83.48).

**Table 3:** Hematology of *O. niloticus* fed with 2% BC supplementation.

Test diets	BC	RBCs (10^−6^ mm^−3^)	WBCs (10^−6^ mm^−3^)	MCH (pg)	MCHC (%)	MCV (fl)	PCV (%)	PLT	Hb (g/100 ml)
I	CON	2.43 ± 0.03^e^	6.46 ± 0.56^ab^	20.34 ± 0.57^c^	22.02 ± 0.01^e^	91.85 ± 1.22^c^	20.04 ± 0.04^c^	52.34 ± 0.36^e^	5.78 ± 0.57^cd^
II	CSBC	3.21 ± 0.01^b^	7.46 ± 0.59^a^	26.01 ± 1.10^a^	27.64 ± 0.03^b^	94.49 ± 1.01^b^	25.87 ± 0.02^a^	70.62 ± 0.97^b^	7.32 ± 0.58^ab^
III	WSBC	3.01 ± 0.03^c^	7.38 ± 0.55^a^	25.00 ± 1.00^ab^	25.02 ± 0.01^c^	100.18 ± 0.07^a^	23.34 ± 0.56^b^	64.34 ± 0.36^c^	6.48 ± 0.57^bc^
IV	CCBC	3.42 ± 0.01^a^	7.56 ± 0.48^a^	25.03 ± 1.00^ab^	30.86 ± 0.04^a^	85.19 ± 0.57^d^	26.74 ± 0.05^a^	74.30 ± 0.18^a^	8.18 ± 0.54^a^
V	HWBC	3.42 ± 0.01^g^	5.48 ± 0.55^b^	16.34 ± 0.57^d^	18.45 ± 0.58^g^	83.48 ± 0.58^d^	15.37 ± 0.62^d^	44.04 ± 0.01^g^	4.70 ± 0.59^d^
VI	GWBC	1.99 ± 0.04^f^	6.38 ± 0.55^ab^	19.67 ± 0.57^c^	20.45 ± 0.58^f^	89.81 ± 1.16^c^	19.37 ± 1.17^c^	47.04 ± 0.01f.	5.48 ± 0.57^cd^
VII	GwBC	2.52 ± 0.01^d^	6.78 ± 0.55^ab^	23.34 ± 0.58^b^	24.02 ± 0.01^d^	95.18 ± 1.04^b^	22.04 ± 0.04^b^	56.34 ± 0.36 ^d^	6.61 ± 0.41^bc^

RBC = Red Blood Cell, MCH = Mean corpuscular hemoglobin, WBC = White blood cell, MCHC = Mean corpuscular hemoglobin concentration, PCV = Packed cell volume, PLT = Platelet, MCV = Mean corpuscular volume, Hb = hemoglobin concentration, Biochar (BC), Control (CON, TD- I), cotton stick BC (CSBC, TD-II), wheat straw (WSBC, TD-III), corn cob (CCBC, TD-IV), house waste (HWBC, TD-V), grass waste (GWBC, TD-VI), and green waste (GwBC, TD-VII). ^a–g^Means of columns with different superscripts possess a significant difference (*p < *0.05) and the data comprises the means of 3 replicas.

### Mineralization

The mineral status of each test diet differed markedly from that of the other (Table 4). The mineral digestibility data also revealed that the CCBC (TD-IV) had high digestibility coefficient values when compared to the other test diets. Diet TD-IV showed the best digestion values for Ca (56.81%), Na (52.61%), K (61.07%), P (66.32%), Fe (68.87%), Cu (66.21%), and Zn (68.92%) when given to *O. niloticus* fingerlings whereas lowest values of all these minerals were observed by HWBC (TD-V).

**Table 4:** Mineral status of *O. niloticus* fed with 2% BC supplementation.

Test diets	BC	Ca	Na	P	Cu	Zn	Fe	K
I	CON	36.15 ± 3.1^c^	33.84 ± 0.51^ab^	38.99 ± 3.12^d^	48.52 ± 2.72^e^	43.75 ± 1.67^e^	49.55 ± 1.1^e^	39.43 ± 0.52^bc^
II	CSBC	50.79 ± 2.5^b^	26.56 ± 13.6^b^	58.04 ± 0.44^b^	61.55 ± 2.20^b^	58.15 ± 0.85^b^	63.52 ± 0.6^b^	47.64 ± 14.78^ab^
III	WSBC	43.95 ± 1.1^b^	31.88 ± 7.28^ab^	54.89 ± 0.89^b^	57.53 ± 0.50^c^	53.91 ± 1.04^c^	60.70 ± 0.5^c^	46.27 ± 8.27^abc^
IV	CCBC	56.81 ± 1.0^a^	52.61 ± 3.61^b^	66.32 ± 0.35^a^	66.21 ± 1.17^a^	68.92 ± 0.74^a^	68.87 ± 1.6^a^	61.07 ± 3.53^a^
V	HWBC	15.48 ± 3.7^e^	19.17 ± 6.63^b^	33.16 ± 2.91^e^	40.02 ± 3.20^g^	33.98 ± 1.94^g^	41.27 ± 1.7^g^	25.89 ± 5.03^c^
VI	GWBC	23.82 ± 3.6^d^	27.14 ± 2.35^b^	33.92 ± 1.80^e^	40.63 ± 1.85^f^	37.93 ± 1.02^f^	44.50 ± 1.4^f^	26.58 ± 8.60^c^
VII	GwBC	47.84 ± 0.6^b^	17.89 ± 9.49^b^	49.36 ± 0.65^c^	51.54 ± 1.04^d^	51.42 ± 0.21^d^	51.24 ± 0.7^d^	52.47 ± 0.29^ab^

Biochar (BC), Control (CON, TD- I), cotton stick BC (CSBC, TD-II), wheat straw (WSBC, TD-III), corn cob (CCBC, TD-IV), house waste (HWBC, TD-V), grass waste (GWBC, TD-VI), and green waste (GwBC, TD-VII). ^a–g^Means of columns with different superscripts possess a significant difference (*p < *0.05) and the data comprises the means of 3 replicas.

## Discussion

Identifying the ideal feed formulation is a primary need in aquaculture. Animal based diets are considered the best sources of protein like FM but with the passage of time, prices of FM or animal based diets are increasing. The benefits of using plant based diet as a dietary additive to enhance overall performance of tilapia (*O. niloticus*), have drawn more attention in recent years. This research evaluated the effects of using BC derived from various sources, on growth, nutrient absorption, carcass composition, hematology and mineral status of *O. niloticus*. Some earlier research have reported beneficial findings of adding BC supplements in several types of livestock, including goats, pigs, poultry, and cattle^52–54^.

Interestingly, substituting 2% CCBC in SFM-based diet yielded the greatest growth indices in tilapia. In another study, the growth parameters of *O. niloticus* were significantly improved after 8 weeks of supplementation with activated charcoal at a rate of 7.0 g/kg^55^. The incorporation of activated charcoal into aquafeeds has been shown to adsorb and remove gases and contaminants from the gastrointestinal tract, thereby optimizing nutrient absorption and utilization, which is a key factor contributing to improved fish growth and feed efficiency. Likewise, with the results for *Pangasius hypophthamus*^56^, our study indicated the same conclusion. In this research, bamboo charcoal (2%) when fed to *P. hypophthamus* showed an increased growth performance. In a study conducted by Najmudeen et al.^57^, it was shown that *O. mossambicus* showed a notable increase in both weight and length when given *Eichhornia crassipes* BC at concentrations of 0.5% and 1%. Maximum growth rate was observed in fingerlings fed 1% BC. Moreover, improvements in body weight, FCR, and survival rate were noted in *Plotosus lineatus* (catfish) and *Salmo trutta* (trout) when 2% bamboo BC and 0.2/kg were added to their diets, respectively^56^. Furthermore, it was reported by Michael et al.^58^ that substitution of 3% commercial wood charcoal increased the growth performance (WG:11 g, FCR: 1.07, SGR: 3.89 and PER: 2.89) of red tilapia juveniles.

Current research revealed that except TD-V supplementation of 2% different types of BCs improved the nutrient digestibility of *O. niloticus.* It has been shown that adding BC to cattle feed improves the animals' production, health, and efficiency of nutrient intake^59^. According to Khalid et al.^60^, the addition of 2 mg/kg of poultry waste BC improved the digestion of CP (75.92%), CF (81.90%), and GE (74.84%) in *Catla catla*. Schubert et al.^53^ used two different kinds of BC in accordance with the current research and reported that 2% BC improved the CP, CF, and GE of pigs in addition to having a positive impact on nutrient digestibility. Furthermore, Thu et al.^61^ reported that feeding 4% bamboo charcoal to Japanese flounder resulted in a remarkable increase in body composition (CP: 17.5%, CF: 4.1%, moisture: 72% and ash: 3.5%) and protein digestibility, reaching 89%.

The best results for CP, CF, ash, and moisture in terms of body composition were obtained in the current research with CCBC. Yoo et al.^62^ found that the combination of wood vinegar and charcoal positively correlated with carcass of *Paralichthys olivaceus*, the olive flounder. Thu et al.^61^ found that *P. olivaceus* carcass significantly improved with the use of bamboo charcoal because there was a reduction in ammonia excretion, which raised the quality and protein content of the fish body.

Through the hematological observations, this study concluded that each type of BC supplementation impacted positively on fingerlings except for HWBC (TD-V). The best values of hematological parameters were noted in CCBC (TD-IV). There is little research about the effects of dietary BC on fish hematological indices; conversely, some observations have been reported in cattle and poultry farming. According to Mabe et al.^63^, *Cyprinus carpio* when fed bamboo BC showed no changes in growth indices; however, their serum quality had improved, suggesting better overall fish health. Elghalid^64^ found that hematological traits such as Hb, RBCs and hemocytosis percentage (HCT%) improved when chicks were given diets containing 0%, 1%, 2%, 4%, 6%, or 8% biochar. According to Dim et al.^65^, when BC was given to *Meleagris gallopavo* (turkey) at 5 g kg^−1^, 15 g kg^−1^ and 25 g kg^−1^, 15 g kg^−1^, showed significant improvements in RBC, HCT, Hb, and WBC. BC supplementation in red tilapia plausibly delivered a detoxifying effect that improved hematological parameters and decreased oxidative stress by reducing the absorption of toxins and other potentially harmful substances into the fish gut ^58^.

The current study analysis of the mineral content of body revealed that the TD-IV (CCBC) diet gave the maximum quantity of minerals. When *O. niloticus* fingerlings were fed with the CCBC diet, the best digestibility values of Ca (62.78%), Na (57.03%), K (66.02%), P (67.97%), Fe (70.25%), Cu (67.90%), and Zn (68.92%) were achieved. Biochar has the potential to increase the levels of minerals because of its high cation-exchange capacity, which makes some minerals more readily available^66^.

## Conclusion

In this current study, it was determined that 2% of CCBC delivered positively on growth, carcass, digestibility, hematology and mineralization of *O. niloticus*, except for HWBC (TD-V). Thus, BC can be used as an economical and environmentally sustainable supplement for fish health.

## Data Availability

The data that support the findings of this study are not openly available due to reasons of sensitivity and are available from the corresponding author upon reasonable request.
